# Immunoregulation of Inflammation in Chronic Kidney Disease

**DOI:** 10.1155/2014/897487

**Published:** 2014-02-09

**Authors:** Osamu Takase, Kazuya Iwabuchi, Richard J. Quigg

**Affiliations:** ^1^Department of Advanced Nephrology and Regenerative Medicine, Graduate School of Medicine, University of Tokyo, Hongo 7-3-1, Bunkyo-ku, Tokyo 113-8655, Japan; ^2^Department of Immunology, Kitasato University School of Medicine, 1-15-1 Kitasato, Minamiku, Sagamihara, Kanagawa 252-0374, Japan; ^3^Division of Nephrology, Department of Medicine, State University of New York at Buffalo, School of Medicine and Biomedical Sciences, 875 Ellicott Street, Suite 8030, Buffalo, NY 14203, USA

Chronic kidney disease (CKD) is a commonly encountered condition in clinical practice. Up to 15% of the adult population worldwide is affected by CKD. Patients with CKD are at a high risk of developing hypertension and other cardiovascular diseases, with associated morbidity and mortality. Stage V is end-stage renal disease (ESRD), for which patients require renal replacement therapy with either dialysis or renal transplantation. A patient with ESRD on dialysis has a 50% chance of surviving three years. Overall, CKD is a disease with a tenfold increased incidence over three decades in which affected patients can lose 70% of their life span.

There are many underlying etiologies of CKD, including diabetes, hypertension, primary and secondary glomerulonephritis/vasculitis, and tubulointerstitial nephritis. Irrespective of initiator, there appears to be an important role for inflammation in CKD. In fact, patients with CKD are frequently treated nonspecifically with immunosuppressive and/or antihypertensive agents. Unfortunately, there have not been new treatment regimens for CKD induced by various underlying causes.

This special issue covers the broad topic of immunoregulation of inflammation in CKD. Four review articles and two research articles discuss the mechanism of inflammation in animal models of lupus nephritis and obstructive uropathy, as well as effects on resident renal cells. It is our hope that these contribute to a better understanding of immunoregulation of inflammation in CKD, which can stimulate development of better therapeutic approaches and provision of optimal care to patients.

In the first paper, “*Mediators of inflammation and their effect on resident renal cells: implications in lupus nephritis*,” S. Yung et al. review immunoregulation in lupus nephritis, an important cause of CKD. They concentrate on interleukin-6 (IL-6), tumor necrosis factor-*α* (TNF-*α*), type I interferons (IFNs) and hyaluronan, as among the most important mediators in this disease. They provide background of the fundamental biology of IL-6, TNF-*α*, type I IFNs, and hyaluronan, followed by their roles in both experimental and human lupus nephritis.

In the second paper, “*LMW heparin prevents increased kidney expression of proinflammatory mediators in (NZB×NZW)F1 mice*,” A. Hedberg et al. have studied effects of low molecular weight (LMW) heparin on lupus nephritis occurring in (NZB×NZW)F1 (B/W) mice. Like in human lupus nephritis, these mice progressively develop CKD. As this occurs, there is increased expression of a diversity of proinflammatory mediators. In their study, they show that LMW heparin specifically lowers CCR2, IL-1*β*, and TLR7 expression. This may be attributable to the ability of LMW heparin to enhance nucleosomal degradation and/or binding to glomerular sites as components of immune complexes.

In the third paper, “*Interactions between cytokines, congenital anomalies of kidney and urinary tract and chronic kidney disease*,” A. C. S. Silva et al. reviewed the relative roles for cytokines and chemokines in the pathophysiology of congenital anomalies of the kidney and urinary tract and how they can affect progression of CKD. They include experimental and clinical evidence to show that urine measurements of cytokines could prove useful as predictors of urinary tract obstruction and renal scarring.

In the fourth paper, “*MicroRNAs implicated in the immunopathogenesis of lupus nephritis*,” C. B. Chafin and C. M. Reilly provide a review that addresses a current and important topic: the role of microRNAs in the pathogenesis of lupus nephritis. The authors collated a large amount of available data regarding the potential role of microRNAs as therapeutic targets and how these might underlie potential treatment strategies for this important disease.

In the fifth paper, “*Inflammatory chemokine expression via toll-like receptor 3 signaling in normal human mesangial cells*,” H. Tanaka and T. Imaizumi summarize their experimental results regarding signaling pathways in human mesangial cells activated upon treatment with a synthetic analogue of viral dsRNA. The signaling pathways activated through TLR3 in mesangial cells may be proinflammatory. The relevance includes effects of viral and “pseudoviral” infections on existing CKD, as well as pathogenic mechanisms that may underlie primary glomerulonephritis.

In the sixth paper, “*Contrasting effects of systemic monocyte/macrophage and CD4* ^*+*^ *T cell depletion in a reversible ureteral obstruction mouse model of chronic kidney disease*,” L. Chaves et al. show that depleting macrophage and CD4^+^ T cells had distinct effects on manifestations of CKD in a reversible model of unilateral ureteral obstruction (rUUO). Based on these results, the authors concluded that modulation of immune cells during injury and repair altered the development of CKD in the rUUO model. Their rUUO model is unique, and findings in this study provide interesting clues to the mechanisms of CKD progression.

We are certain that the readers of this special issue will find several interesting points of discussion in the published papers. We hope these papers can stimulate further experimentation to dissect the immunoregulation of inflammation in CKD and allow development of new therapeutic strategies for CKD.

